# Investigation on Fluorescence Quenching Mechanism of Perylene Diimide Dyes by Graphene Oxide

**DOI:** 10.3390/molecules21121642

**Published:** 2016-11-30

**Authors:** Yuzhen Zhao, Kexuan Li, Zemin He, Yongming Zhang, Yang Zhao, Haiquan Zhang, Zongcheng Miao

**Affiliations:** 1 Department of Applied Statistics and Sciences, Xijing University, Xi’an 710123, China; zyz19870226@163.com (Y.Z.); likexuanvip@163.com (K.L.); zhangyongming@xijing.edu.cn (Y.Z.); zhaoyang@xijing.edu.cn (Y.Z.); 2 Department of Materials Science and Engineering, College of Engineering, Peking University, Beijing 100871, China; 3 State Key Laboratory of Metastable Materials Science and Technology, Yanshan University, Qinhuangdao 066004, China; zeminhe315@126.com

**Keywords:** electron transfer, perylene diimide dyes, fluorescence quenching mechanism, graphite oxide

## Abstract

Perylene diimide derivatives were used as probes to investigate the effect of the molecular structures on the fluorescence quenching mechanism in a perylene diimide/graphene oxide system. The electrons transferred from the excited state of dyes to the conductive band of graphene oxide with different concentrations were determined by fluorescence spectra. The results indicated that the quenching efficiency of perylene diimides by graphene oxide was not only dependent on the difference between the lowest unoccupied molecular orbital level of dyes and the conduction band of the graphene oxide, but also mainly on the difference in the molecular structures.

## 1. Introduction

Graphene oxide (GO) has been widely used in DNA detection and other analytes, such as Ag^+^, ATP, etc. [1,2,3]. It is very important to note that GO-based sensors have to meet one prerequisite which is that the analyzed target does not nonspecifically adsorb onto the surface under the experimental conditions; otherwise, the sensing performance will be dramatically degraded or the sensor will fail to work [4]. Thus, the molecular structure of the analyzed target determines its sensitivity [5,6]. Supur M et al. reported fast photo-induced charge separation in perylene diimide–graphene oxide (TAIPDI-GO) hybrid layers [7]. Significant spectral changes in the absorption of TAIPDI-GO and emission quenching indicate π–π interactions between the π-surfaces of perylene diimide (PDI) and GO [7]. This typically favors photo-induced electron transfer due to the very close distance of the noncovalent materials with large π-surfaces [8,9,10,11]. As is well known, the interaction between conjugated molecules and GO sheets mainly includes π–π interactions and electrostatic attraction between atoms, etc. [12]. The relation between the chemical structure of the analyzed target and the fluorescent property of the sensor based on GO is still not clear. Fortunately, our groups have recently synthesized a series of perylene diimide derivatives, which possessed only slight differences in molecular structures and energy levels [13]. This provided sufficient resources for us to investigate the influence of molecular structure on the fluorescence quenching mechanism of perylene diimide derivatives by GO.

Thus, in this article, four different dye molecules, namely *N*,*N*′-diethylhexyl-1-bromo-perylene diimide (SBrPDI), *N*,*N*′-diethylhexyl-1,7-dibromo-perylene diimide (DBrPDI), *N*,*N*′-diethylhexyl-1-bromo-7-pentafluorophenoxyl-perylene diimide (SFPDI), *N*,*N*′-diethylhexyl-1,7-dipentafluoro-phenoxyl-perylene diimide (DFPDI) in *N*,*N*′-dimethylformamide (DMF) were used as probes to investigate the electron transfer from the excited state of the dyes to the conductive band of GO. The relationship between the molecular structures of PDIs and its fluorescence quenching mechanism was investigated.

## 2. Results and Discussion

### 2.1. The UV-Vis Studies of GO and Dyes

**Figure 1:**
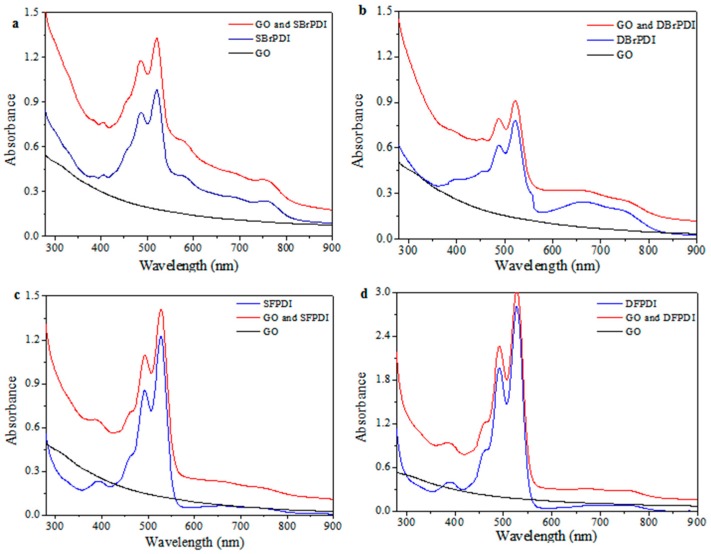
Absorption spectra of perylene diimide derivatives (PDIs), the 1:1 mixture with mass ratio of PDIs/GO and GO in *N*,*N*′-dimethylformamide (DMF) (Concentration of PDIs, C = 10^−5^ M). DBrPDI: *N*,*N*′-diethylhexyl-1,7-dibromo-perylene diimide; DFPDI: *N*,*N*′-diethylhexyl-1,7-dipentafluoro-phenoxyl-perylene diimide; GO: Graphene oxide; SBrPDI: *N*,*N*′-diethylhexyl-1-bromo-perylene diimide; SFPDI: *N*,*N*′-diethylhexyl-1-bromo-7-pentafluorophenoxyl-perylene diimide.

The absorption peaks of PDI are at 490 nm and 525 nm, respectively. The absorption peak of PDIs in the 1:1 mixture (mass ratio of PDIs and GO) does not show any shift compared to that of pure PDIs in DMF (Figure 1). Moreover, the absorption spectra of the 1:1 mixture of PDI and GO are almost identical to the sum of the absorption spectra of isolated GO and isolated PDI with the same concentrations. All these results suggested that there was no interaction between GO and PDIs at ground state in DMF [14].

### 2.2. The Fluorescence Studies of Dyes

**Figure 2:**
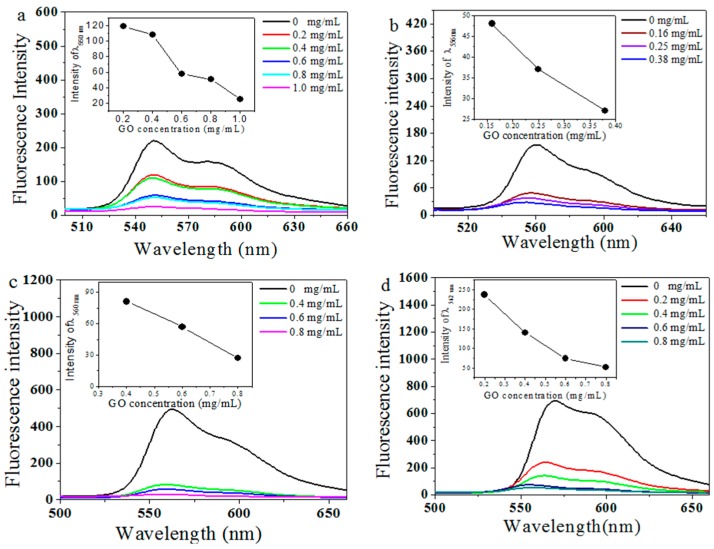
Fluorescence spectra of dyes (10^−5^ mol/L) with different GO concentrations; SBrPDI (**a**); DBrPDI (**b**); SFPDI (**c**); DFPDI (**d**) in *N*,*N*-dimethylformamide. Insert: the linear relationship of the corresponding dye fluorescence and GO concentration (mg/mL) (excited wavelength: 525 nm).

Fluorescence spectra of all dyes (10^−5^ mol/L) with different concentrations of the GO are shown in Figure 2. Emission peaks appeared at 550 nm, 560 nm, 562 nm and 571 nm for SBrPDI, DBrPDI, SFPDI and DFPDI, respectively. It can be seen that the photoluminescence (PL) intensity of all dyes gradually decreased when GO was added into the dye solution. Moreover, upon the concentration of GO increasing to a high value, the PL intensities were almost quenched.

**Figure 3:**
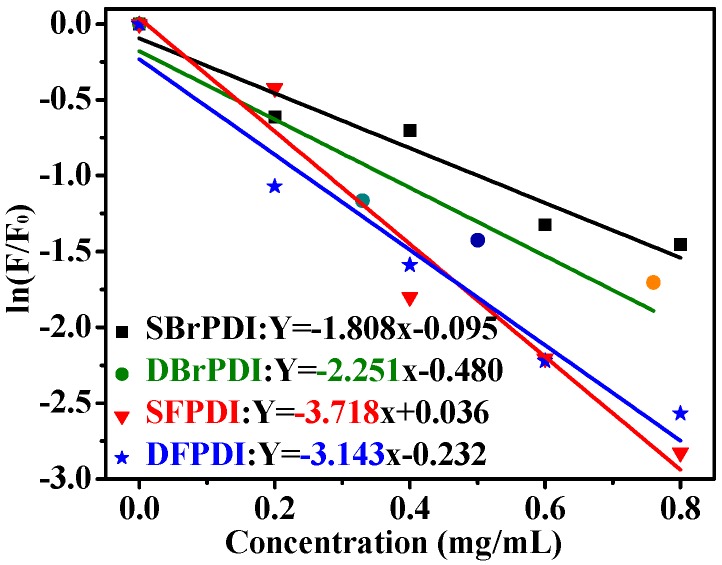
The relationship between quenching efficiency and GO concentration. F: fluorescence intensity of dyes; F_0_: fluorescence intensity after adding GO.

The quenching proportions (QP) of dyes by GO were obtained in Table 1. It can be seen that the QP of SFPDI is the biggest. The fluorescence data were further fitted into linear function curves (Figure 2). The slope of the fitting curves (quenching magnitude, Table 1) was related to the efficiency of the fluorescence quenching. Additionally, a higher slope means a better efficiency of fluorescence quenching. Therefore, the highest slope of SFPDI indicated that the fluorescence quenching efficiency of GO on SFPDI dye was higher than on the other dyes (Figure 3).

### 2.3. The Mechanism of Fluorescence Quenching

Essentially, fluorescence quenching is ascribed to the transfer from the quenching of the electron singlet excited state back to the ground state through a nonradioactive transition by either energy or electron transfer in association with the property of the fluorophore, conjugation mode, and local environment. However, there was no overlap between the absorption spectra of GO and the emission band of the dyes and the GO did not show any PL emission peaks in the range of 500–600 nm; thus, the fluorescence quenching of the dyes by GO went through an electron transfer from the excited PDIs (excited state of PDIs) to the semi-conductive GO [15,16].

**Figure 4:**
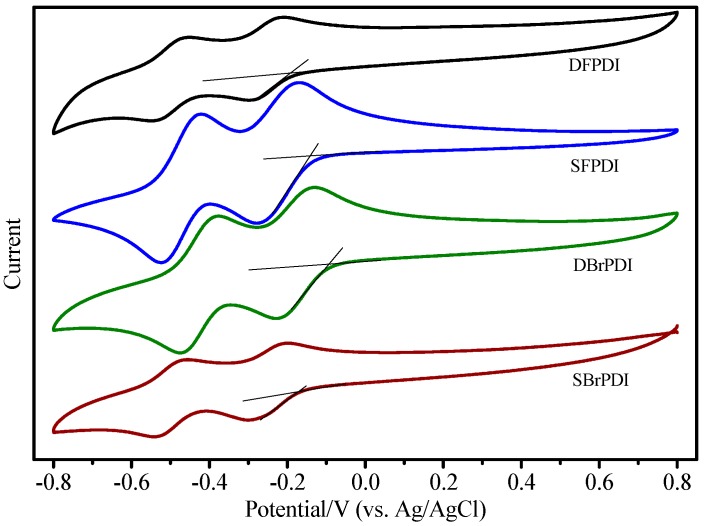
Cyclic voltammetry of dyes (C = 10^−5^ M) recorded in DMF containing 0.1 M *n*-Bu_4_NPF_6_ at 0.1 Vs^−1^.

The dependence of the absolute PL emission quenching of dyes on the GO concentration further confirmed this conclusion (PL intensities of dyes are strongly dependent on the GO concentration) [14]. The GO itself is semi-conductive and its work function (−4.7 eV) [17,18], is lower than the lowest unoccupied molecular orbital (LUMO) level of the dyes. The LUMO levels of SBrPDI, DBrPDI, SFPDI, DFPDI dyes calculated from the cyclic voltammograms (vs. AgCl/Ag, Figure 4) of dyes were −4.57 eV, −4.65 eV, −4.59 eV, −4.55 eV, respectively; thus, the electrons were able to transfer from the excited state of the dyes to the conduction band of GO. The energy levels of SFPDI and DFPDI are higher than that of the DBrPDI dye (Figure 2b). Therefore, the electron transfer from the excited SFPDI and DFPDI dyes to GO is the more effective than from DBrPDI.

**Figure 5:**
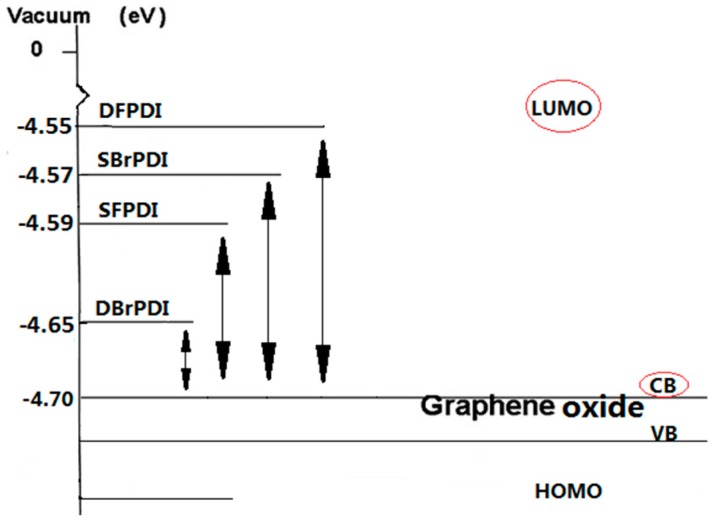
Schematic energy-level diagram of GO relative to the level of the excited-state dyes. CB: conduction band, VB: valence band, HOMO: highest occupied molecular orbital.

For Br-substituted dyes (SBrPDI, DBrPDI), though the LUMO energy level of SBrPDI is higher than that of DBrPDI (Figure 5), the quenching efficiency of GO on PDIs was the opposite. Our results show that the quenching efficiency of GO on DBrPDI was higher than that of SBrPDI. This reason was that the electrostatic attraction in the PDIs/GO system played a more important role because of the introduction of the Br atom [19]. The electrostatic attraction between DBrPDI and the hydrogen atom of the hydroxyl or carboxyl of GO was greater than that of the hydrogen atom of SBrPDI with the hydrogen atom of the hydroxyl or carboxyl of GO. Similarly, the results of other systems were the same; that is, the quenching efficiency of PDIs is not only dependent on the difference between the LUMO energy level of the dye and the conduction band of GO, but also mainly on the molecular structures of the dyes.

## 3. Materials and Methods

### 3.1. Materials

**Figure 6:**
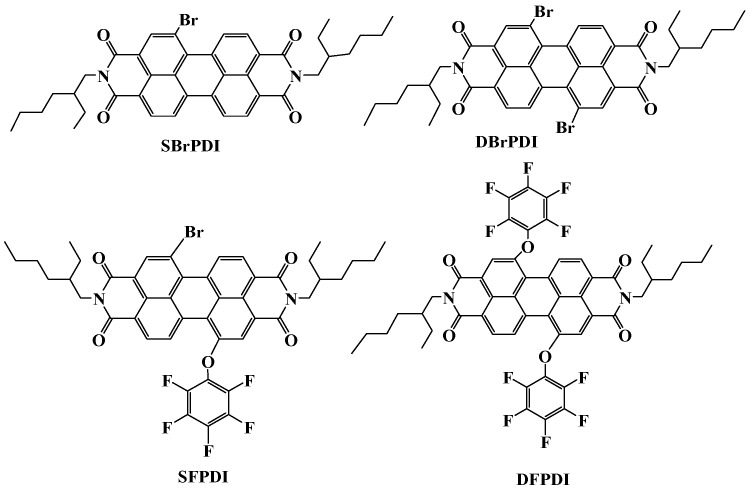
Molecular structures of the dyes SBrPDI, DBrPDI, and SFPDI, DFPDI.

SFPDI, DFPDI, SBrPDI, DBrPDI were prepared by our group (Molecular structures are shown in Figure 6) [13]. Graphene oxide sheets were purchased from Nanjing Cangji Technology Co., Ltd. (Thickness: <1 nm; Diameter Price: 100 nm~5 μm; Carbon atom/Oxygen atom: 1~3). All chemicals, including DMF were obtained from Aladdin with analytical grade.

### 3.2. Characterization

Ultraviolet-Visible (UV-Vis) spectra were recorded using a Perkin Elemer Lambda 35. Fluorescence Spectroscopy was recorded on a Perkin Elmer RF-5301PC spectro-photometer using 1 cm path length quartz cells. All the experiments were performed at room temperature.

### 3.3. Experiment

First, the purchased brown GO in DMF was ultrasonically vibrated at 35 Hz for 60 min to obtain a brown stable GO dispersion at room temperature. Then, the synthesized PDIs in DMF were ultrasonically vibrated at 35 Hz for 60 min to obtain brown stable PDIs dispersion at room temperature. And the GO dispersion (5.0 mL) was mixed with 5.0 mL of 4 mg/mL PDIs DMF solution, and the mixed solution was then stirred at 50 °C for 20 min. The stable black dispersion was obtained.

## 4. Conclusions

The fluorescence quenching mechanism was ascribed to the electron transfer from the excited state of the dyes to the conductive band of GO. The electron transfer mechanism from the dyes to GO was fully discussed. The results indicated that the quenching efficiency of PDIs by GO not only depended on the difference between the LUMO energy level of dyes and the conduction band of graphene oxide, but also mainly on the difference in the molecular structures.

## Figures and Tables

**Table 1:** Fluorescence quenching parameters of dyes and GO (0.8 mg/mL).

System	SBrPDI + GO	DBrPDI + GO	SFPDI + GO	DFPDI + GO
Quenching magnitude	1.808	2.251	3.718	3.413
Quenching proportion	0.8615	0.8409	0.9227	0.8952
